# Proceedings of the Indiana State Dental Society

**Published:** 1877-08

**Authors:** J. A. Turner


### PROCEEDINGS OF THE INDIANA STATE DENTAL SOCIETY.

The nineteenth annual meeting of the Indiana Dental Society was held at Fort Wayne on the 26th of June.

The meeting was called to order at 10 o'clock A. M. by the President Dr. Isaac Knapp.

The Committee on Dental Legislation was granted further time.

The first subject taken up was Exposed Pulps, and a paper by Dr. F. E. Eddleman was read by Dr. Wells. Upon a discussion of the subject, Dr. Cushing of Chicago, stated that his first experience was that Dr. Atkinson's method in the use of the oxy. chloride of zinc was formerly regarded as quite successful, but for two years past had been unsatisfactory.

Dr. Merit Wells stated that in some cases Dr. Craven's method in the use of the lacto-phosphate of lime had been successful where a new deposit of dentine had covered the Exposed Pulp.

The next subject, "Treatment of Exposed Pulp and Aveolar Abscess," was taken up, and papers were read by Drs. J. A. Turner and S. C. Carter. The various methods and materials for filling were referred to, and Hill's stopping, gutta percha, oxy. chlroide of zinc and gold were all found to be successful agents in the preservation of the roots of pulpless teeth. Dr. Smith of Cincinnati had found that Salycilic Acid acted upon the dentine by disintegrating it.

At the beginning of the afternoon session the question was asked, "How shall I remedy the condition where the tooth is loose from absorption of the alveolar process?" Dr. Cushing stated that his experience was that it was caused by deposit and by removing the cause the cure followed.

Dr. H. S. Chase's paper on Plastic Dentistry was then read and discussed. The paper took the grounds that a material must be found by the coming dentist that shall be plastic. Dr. Cushing disagreed with the premises of the paper, because the experiments had all been made out of the mouth, and that we must not abandon the tried for the untried and unproved Dr. Smith thought that the coming dentist would be a plastic man.

Upon reassembling at 7:45 P.' M. the papers of Dr Chase on Plastic Fillings was resumed in connection with the subject:

### TIN AND AMALGAM FOR FILLING TEETH.

Dr. Smith stated that oxy-chloride filling can be made very hard by rubbing into the oxy-chloride magnesia or soapstone—French chalk—with a hot iron.

Dr. Keely advocated the use of tin in cases of children's teeth.

Dr. Knapp uses amalgam in children's teeth in many cases where the patient is difficult of control.

### THIRD-DAY, MORNING SESSION.

The care of children's teeth was taken up, and the question was asked: What shall be done in the case of an eight year old child whose deciduous centrals were gone and one permanent central was presenting proximal surface to front?

Dr. Cushing would use platinum band with gold bar soldered to same and so bring the tooth to normal position. Where tenison and the help given by the lips, tongue and muscles can be called to our help, it were preferable. Dr. Keely had learned that gold in deciduous teeth was unnecessary, and preferred amalgam, and made it a rule never to extract the molars until full time for the eruption of permanent. When upper centrals are in too far, makes a plate, passing it over the back teeth, and a band under the lip outside the teeth and ligatures over teeth and so brings them out.

On the subject "Replantation of Teeth," Dr. Keely would not advise extraction and filling in order to replant in cases where the cavity seems to be inaccessible.

Dr. Taft had found that there were some cases that seemed to be favorable for replanting: for instance where aveolar abscess was the condition, extraction and replantation was the most successful method of treatment. His experience was that the removal of abscess and the entire removal of periosteum did not prevent a firm attachment of the replanted tooth. Immediately upon extraction places in the socket a pledget of cotton saturated with extracts of Hammamillia, to prevent coagulation of blood; fill the cavity of decay, the pulp chamber and canals, then replace the tooth.

A tooth may remain out of the socket, with impunity sufficiently long for any ordinary operation of filling.

Does not regard material used for filling root as important so that the material used does not decompose. Usually use in extraction a local anæsthetic.

At the opening of the afternoon session, Dr. C. Sihler of Cleveland, read a paper upon Microscopy, explaining his theories by charts and drawings and anotomical preparations. The paper was elaborate and very suggestive. His views differ from Tomes and Koeliker and others on the structure of dentine.

The Association passed a vote of thanks, and requested that he allow the publication of the paper.

Dr. Taft then requested Dr. Sihler's opinion as to why dentine was sensative?

Dr. Sihler stated that he did not see the necessity for nerve tissue in tubules of dentine, but rather that the sensativeness might result without nerves in dentine, just as an impression is made by pricking the skin.

It is no proof of a nerve in a certain locality because it is sensative at that point.

EVENING SESSION.

The newly elected president, Dr. T. S. Hacker in the chair. Upon motion it was ordered that $50 be donated to the "Ohio College of Dental Surgery."

The Treatment of Sensative Dentine was taken up. Dr. Taft had found that the condition varied, owing to the many causes, which produced the same. Malarial districts were prolific of the condition. Sensativeness in the cavity in all its parts, is often found where only a lamina of the dentine is sensative. The sensativeness may be removed by a few scrapes of the burr or excavator. Where the sensativeness is so deep excavations will not removed it, therapeutic agents must be used to remedy.

the condition. Dryness is a very great help in excavating sensative dentine.

Systematic treatment is sometimes demanded, either by the physician or dentists. When you can not fill immediately without danger, fill with some non-conducter, as Hill's stopping, and wait for a more favorable condition, but the patient should be treated systemically.

Dr. Stellwager's plan of a piece of hard wood with hot end inserted into cavity was dangerous because of devitalization.

Dr. Smith had tried Dr. Stellwager's method, and in one case had found relief.

The method was to apply the first warm, and then warmer, until the live coal can be applied without danger or pain. Orange wood was the best because it retains a firm ash and gives a good point to apply to the cavity. Discussion followed by Dr. Cushing who wished to emphasize the fact that systemic treatment had much to do with the sensativeness of dentine. Uses sometimes oil of peppermint.

The President then announced that the delegates to the American Dental Association, could be supplied with certificates by the Secretary.

Adjourned to meet at Indianapolis the last Tuesday of June, 1878.

J. A. TURNER.

*Secretary, Pro tem.*

